# Antibacterial Isoquinoline Alkaloids from the Fungus *Penicillium Spathulatum* Em19

**DOI:** 10.3390/molecules24244616

**Published:** 2019-12-17

**Authors:** Christina Nord, Jolanta J. Levenfors, Joakim Bjerketorp, Christer Sahlberg, Bengt Guss, Bo Öberg, Anders Broberg

**Affiliations:** 1 Department of Molecular Sciences, Uppsala BioCentrum, Swedish University of Agricultural Sciences, P.O. Box 7015, SE-750 07 Uppsala, Sweden; Christina.Nord@slu.se (C.N.); Jolanta.Levenfors@slu.se (J.J.L.); Joakim.Bjerketorp@slu.se (J.B.); 2 Ultupharma AB, Södra Rudbecksgatan 13, SE-752 36 Uppsala, Sweden; bo.oberg1@gmail.com; 3 Medivir AB, P.O. Box 1086, SE-141 22 Huddinge, Sweden; christer.sahlberg@gmail.com; 4 Department of Biomedical Sciences and Veterinary Public Health, Swedish University of Agricultural Sciences, P.O. Box 7036, SE-750 07 Uppsala, Sweden; Bengt.Guss@slu.se; 5 Department of Medicinal Chemistry, Uppsala University, P.O. Box 574, SE-751 23 Uppsala, Sweden

**Keywords:** antibiotic resistance, antibacterial secondary metabolites, secondary metabolism, *Penicillium*, isoquinoline alkaloids, dehydroalanine

## Abstract

In the search for new microbial antibacterial secondary metabolites, two new compounds (**1** and **2**) were isolated from culture broths of *Penicillium spathulatum* Em19. Structure determination by nuclear magnetic resonance and mass spectrometry identified the compounds as 6,7-dihydroxy-5,10-dihydropyrrolo[1,2-*b*]isoquinoline-3-carboxylic acid (**1**, spathullin A) and 5,10-dihydropyrrolo[1,2-*b*]isoquinoline-6,7-diol (**2**, spathullin B). The two compounds displayed activity against both Gram-negative and -positive bacteria, including *Escherichia coli*, *Acinetobacter* *baumannii*, *Enterobacter* *cloacae*, *Klebsiella* *pneumonia*, *Pseudomonas* *aeruginosa*, and *Staphylococcus* *aureus*. Compound **2** was more potent than **1** against all tested pathogens, with minimal inhibitory concentrations down to 1 µg/mL (5 µM) against *S. aureus*, but **2** was also more cytotoxic than **1** (50% inhibitory concentrations 112 and 11 µM for compounds **1** and **2**, respectively, towards Huh7 cells). Based on stable isotope labelling experiments and a literature comparison, the biosynthesis of **1** was suggested to proceed from cysteine, tyrosine and methionine via a non-ribosomal peptides synthase like enzyme complex, whereas compound **2** was formed spontaneously from **1** by decarboxylation. Compound **1** was also easily oxidized to the 1,2-benzoquinone **3**. Due to the instability of compound **1** and the toxicity of **2**, the compounds are of low interest as possible future antibacterial drugs.

## 1. Introduction

The global spreading of bacterial resistance to existing antibiotics is a severe and escalating problem to humanity, as frequently pointed out by many authorities and organizations, e.g., the report from WHO’s Global Antimicrobial Resistance Surveillance System (GLASS) [1]. Effective treatment of bacterial infections is one cornerstone in modern society, and the spread of antibiotic resistance will seriously limit the possibilities to treat simple infections. Antibiotic resistance will also severely limit surgery and organ transplantation [2]. Due to the spread of antibiotic resistance, there is a great need for new antibiotics, and in particular, drugs with new mechanisms of action. Already in 2010, the Infectious Diseases Society of America (IDSA) launched the “10 × ‘20 Initiative”, which seeks a global commitment for the development of ten new systemic antibiotic drugs by 2020 [3]. The US Food and Drug Administration (FDA) has, since 2010, approved 20 new molecular entities for the treatment of bacterial infections [4]. Of these, however, only the macrolide antibiotic fidaxomicin (FDA approved in May 2011) has a new target, the bacterial RNA polymerase “switch region” [5], whereas the remainder are analogues to existing types of drugs, or combinations with new versions of beta-lactamase inhibitors. Thus, there is a great and unmet need for new antibacterial drugs, and in particular, drugs with new mechanisms of action [6].

The majority of antibiotics used today and in the past are either natural products of microbial origin or developed from such molecules, and microorganisms are still considered as very promising sources for finding new antibacterial secondary metabolites. Among many important microbial taxa for the production of antibacterial compounds, the 300+ species of the genus *Penicillium* have been the source for more than 2000 secondary metabolites [7], as exemplified by benzylpenicillin (penicillin G) [8], the indole alkaloid brevianamide A [9], the polyketide griseofulvin [10], the isocoumarin derivative ochratoxin A [11], and the steroid wortmannin [12].

**Figure 1:**
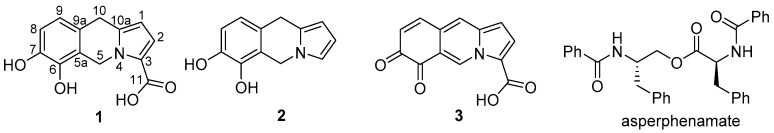
Structures of compounds **1**, **2**, **3**, and asperphenamate.

We are currently searching for new antibacterial compounds produced by fungi and bacteria, in a screening effort at the Swedish University of Agricultural Sciences, with the overall goal of finding candidates for new antibiotics. The present paper describes the isolation and structure determination of two new antibacterial isoquinoline alkaloids, spathullin A and B (**1** and **2**), from *Penicillium spathulatum* Em19, along with one benzoquinone (**3**) formed by oxidation of **1** (Figure 1). Additionally, the antimicrobial activities of **1** and **2** are described, as well as a proposed biosynthetic origin of compound **1**.

## 2. Results and Discussion

### 2.1. Isolation of Compounds

During the screening of bacterial and fungal isolates for the production of antibacterial compounds, the fungus *Penicillium spathulatum* Em19 was found to produce two compounds with activity against both Gram-negative and Gram-positive bacteria. Analysis of antibacterial HPLC-fractions by HPLC-MS suggested that the active compounds have the molecular formulae C_13_H_11_NO_4_ (**1**) and C_12_H_11_NO_2_ (**2**), respectively, indicative of two related compounds differing by CO_2_. Literature search suggested these compounds to be previously undescribed, and the compounds were selected for further characterization. Following culture upscaling, extraction, gradient and isocratic preparative HPLC, 6.3 mg (**1**) and 3.5 mg (**2**) of the respective compounds was isolated.

### 2.2. Compound Identification

**Figure 2:**
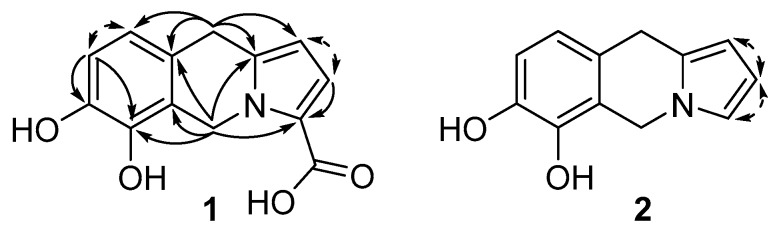
Diagnostic HMBC (single headed arrows) and COSY (dashed double headed arrows) correlations of compounds **1** and **2**.

The molecular formula for compound **1**, C_13_H_11_NO_4_, suggested a degree of unsaturation of nine. Eleven sp^2^ carbons were identified by one-dimensional ^13^C NMR analysis (Table 1 and Figure S2) and ten of these were suggested to be involved in carbon–carbon double bonds and one to be part of a carbonyl group (δ_C_ 162.4), indicating a tricyclic structure for **1**. Data from COSY and HSQC NMR experiments (Figures S3 and S4) enabled the identification of two sp^2^ -CH-CH- spin systems (H-1/H-2, and H-8/H-9, respectively, Figure 1), along with two isolated CH_2_ groups (H_2_-5 and H_2_-10). HMBC data (Figure 2 and Figure S5) showed correlations between the H_2_-10 hydrogens and C-1 and C-9, and two additional sp^2^ carbons (C-9a and C-10a). The H_2_-5 hydrogens also had HMBC correlations to the sp^2^ carbons C-9a and C-10a, as well as to three other sp^2^ carbons (C-3, C-5a and C-6). HMBC correlations were also observed from H-8 to two sp^2^ carbons (C-6 and C-7) and from H-2 to C-3. As judged by their chemical shifts, C-6 (δ_C_ 142.7) and C-7 (δ_C_ 143.5) were suggested to be oxygen linked. These findings are in accordance with a tricyclic core structure containing a catechol moiety and a pyrrole ring connected via two isolated CH_2_ groups forming a central six-membered ring, i.e., a 5,10-dihydropyrrolo[1,2-*b*]isoquinoline alkaloid. The proposed core structure had three open ends: the oxygen atoms at C-6 and C-7, and at C-3 in the pyrrole moiety, and by comparison with the molecular formula, C-6 and C-7 were proposed to be hydroxyl substituted and C-3 to carry a -COOH group. The presence of the latter group was in line with the presence of a carbonyl signal at δ_C_ 162.4 in the one-dimensional ^13^C NMR spectrum (Table 1). Thus, the structure of compound **1** was concluded to be 6,7-dihydroxy-5,10-dihydropyrrolo[1,2-*b*]isoquinoline-3-carboxylic acid (Figure 1), which was given the name spathullin A.

The formula of compound **2**, C_12_H_11_NO_2_, differed from the formula of **1** by CO_2_. The ^1^H and ^13^C NMR data for compounds **1** and **2** are very similar (Table 1), but the ^13^C NMR spectrum of **2** (Figure S7) lacked the -COOH signal of compound 1 (δ_C_ 162.4). Instead, the ^1^H NMR data of **2** (Figure S6) had a new signal at δ_H_ 6.67 (H-3) which formed a sp^2^ -CH-CH-CH- spin system together with H-1 and H-2. Thus, the data suggest that the –COOH group in compound **1** was replaced by a hydrogen in **2**, i.e., compound **2** was 5,10-dihydropyrrolo[1,2-*b*]isoquinoline-6,7-diol, which was called spathullin B (Figure 1).

It was found that compound **1** was spontaneously degraded to **2** in weak acidic solutions (e.g., 0.2% formic acid in water/acetonitrile). With respect to decarboxylation, compound **1** should have properties similar to pyrrole-2-carboxylic acid, which has been found to decarboxylate in acidic water solutions already at pH 2.6 [13], which is similar to the pH of an aqueous 0.2% solution of formic acid.

Compound **1** was also found to partly degrade when stored in acetone-*d*_6_ solutions for a few days at room temperature (ca 3:2 ratio for compounds **1**/**3**). The major degradation product was a brightly purple-colored compound (**3**). Mass spectrometry suggested the molecular formula C_13_H_7_NO_4_ for the compound and NMR spectroscopic investigation, including COSY, HSQC and HMBC experiments (Figures S11–S15), showed that two sp^2^ linked hydrogens (H-5 and H-10) had replaced the two isolated CH_2_ groups and also dramatic shifts of the C-6 and C-7 resonances (δ_C_ 142.7 to δ_C_ 179.1 and δ_C_ 143.5 to δ_C_ 181.4, respectively). These findings suggest that compound **1** had been oxidized to the 1,2-benzoquinone **3**, which was proposed the name spathullin C.

In addition to the new compounds **1**–**3**, the known compound asperphenamate [14] (Figure 1) was tentatively identified by HRMS-data from UHPLC-MS analysis of *P. spathulatum* Em19 culture broths. The production of this compound appears to be general to *P. spathulatum*, as when the species was first described, all 19 investigated isolates of the fungus were found to produce this compound [15], and the compound has also been described to be produced in one further isolate of *P. spathulatum* [16]. In addition to asperphenamate, *P. spathulatum* has been shown to produce an array of different compounds, i.e., perinadine, benzomalvins, breviones, quinolactacin, cyclopenol and related benzodiazepins, as well as a new anthraquinone [15,16], but UHPLC-MS analysis did not suggest that any of these substances were produced by *P. spathulatum* Em19. Among these compounds there are alkaloids of the quinoline, quinazoline and benzodiazepine types, but compounds **1** and **2** are the first isoquinoline alkaloids from this fungus. Other members of the genus *Penicillium* have been described to produce a number of different isoquinoline alkaloids [17,18,19,20,21], but alkaloids containing a pyrrolo[1,2-*b*]isoquinoline structural motif, such as **1** and **2**, have never been found in any fungus or bacterium. Alkaloids with a pyrrolo[1,2-*b*]isoquinoline structural motif have, however, been found in a number of different plant species, e.g., amarbellisine from the plant *Amaryllis belladonna* [22] and fistulosine from the bark of *Ficus fistulosa* [23], but the 5,10-dihydropyrrolo[1,2-*b*]isoquinoline framework of **1** and **2** is hitherto not described in any natural product.

### 2.3. Biosynthetic Considerations

**Figure 3:**
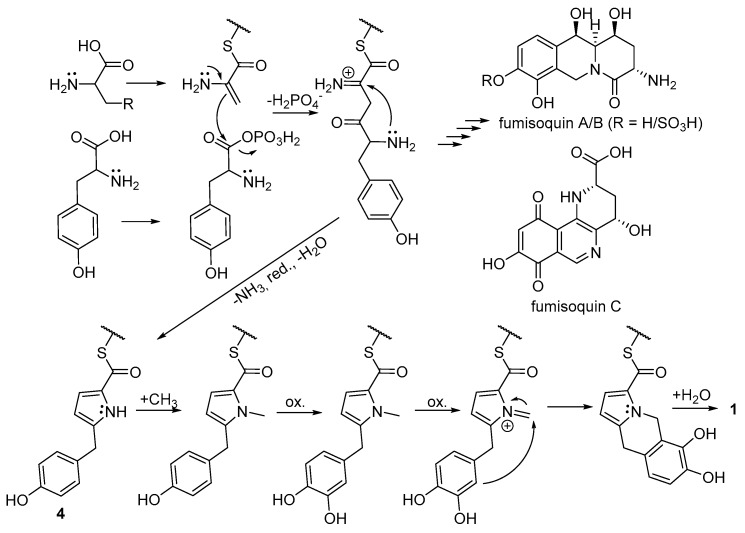
Proposed biosynthesis of compound **1** adapted from the biosynthesis of fumisoquin A–C [24]. R = SH for biosynthesis of compound **1** and R = OH for fumisoquin A–C.

Recently, the biosynthesis of the isoquinoline alkaloids fumisoquin A–C, by the fungus *Aspergillus fumigatus*, was described by Baccile et al. [24] to be performed by enzymes encoded by NRPS-like genes. These authors also demonstrated that fumisoquin A–C were constructed from the amino acids serine, tyrosine and methionine, and they proposed that a carbon-carbon bond was formed between the serine and tyrosine residues via a nucleophilic attack from dehydroalanine onto a presumed tyrosine phosphate ester (Figure 3). Furthermore, Baccile et al. [24] described a shunt metabolite (compound **4** in Figure 3 after thio ester hydrolysis), which is similar to compound **1**, but lacks C-5 and the central six-membered ring as well as OH-6. This suggests that compound **1** may be formed by a similar enzymatic machinery, as outlined in Figure 3, including *N*-methylation of the pyrrole ring of **4**, two oxidation steps, ring-closure from the nucleophilic catechol ring to the iminium moiety, and hydrolysis of the thio ester. To investigate this, *P. spathulatum* Em19 cultures were fed with 1-^13^C-serine, 1-^13^C-tyrosine or ^13^CH_3_-methionine. A subsequent UHPLC-MS analysis showed that ^13^C label from tyrosine and methionine indeed were incorporated into **1**, suggesting that C-1, C-10a, C-10, C-9a, C-9, C-8, C-7, C-6 and C-5a, originate from tyrosine, and C-5 from methionine (Figure 1 and Figure 3). However, ^13^C label from serine was not incorporated into **1**, leaving the origin of C-2, C-3 and C-11 unaccounted for. Dehydroalanine is present in a number of natural products, e.g., nosiheptide [25] and nisin A [26], and has, to the best of our knowledge, only been described to originate from serine residues [27,28], but e.g., cysteine and alanine are also possible precursors of dehydroalanine. Additionally, pyruvate could be the origin of these three carbons, via nucleophilic attack from the corresponding enolate, and these carbons could also have a polyketide origin. To test these possibilities, Em19 cultures were fed with 1-^13^C cysteine, 1-^13^C alanine, sodium 1-^13^C pyruvate or sodium 1/2-^13^C acetate, and were then analysed by UHPLC-MS. These analyses show that ^13^C label from cysteine was incorporated into **1**, but not from alanine or pyruvate. Additionally, both sodium 1- and 2-^13^C acetate were incorporated into **1**, but only when ^13^C acetate was added from the start of cultures, and not when fed as a pulse to a culture which was actively producing **1**, as indicated by UHPLC-MS. These findings indicate that C-2, C-3 and C-11 originate from cysteine, and that the label incorporated from ^13^C acetate, when added from the start of a culture, is likely to be via the biosynthesis of cysteine from ^13^C acetate, and not via a direct polyketide-like pathway. In conclusion, the biosynthesis of compound **1** is proposed to proceed as outlined in Figure 3, i.e., from cysteine derived dehydroalanine, tyrosine and methionine.

Dehydroalanine residues have previously been shown to be involved in bond-formation in peptides, in the posttranslational formation of the thioether amino acid lanthionine of nisin type of peptides, via a Michael-type addition of a nucleophilic cysteine sulfhydryl group onto the electrophilic α,β-unsaturated dehydroalanine [29]. In contrast, the reaction mechanism proposed for fumisoquin A–C [24] and for compound **1** depends on the nucleophilic properties of the enamine type moiety of dehydroalanine, which enables carbon–carbon bond formation to an electrophilic tyrosine moiety. These two examples illustrate the dual nature of dehydroalanine—it can act both as an electrophile and as a nucleophile in secondary metabolite biosynthesis.

As mentioned above, compound **2** forms spontaneously from **1** in weakly acidic water solutions, and it is suggested that the formation of compound **2** also is a spontaneous process in culture broths. Compound **2** also incorporated ^13^C from 1-^13^C tyrosine and ^13^CH_3_ methionine, but not from 1-^13^C cysteine, in line with the proposed formation of **2** by decarboxylation of compound **1**, which results in the loss of the ^13^C label from 1-^13^C cysteine.

### 2.4. Biological Activity of Compounds **1** and **2**

Both compounds showed antibacterial activity against Gram-negative as well as Gram-positive bacterial strains (Table 2). For all tested bacteria, compound **2** displayed the lower minimal inhibitory concentration (MIC) of the two compounds and for both **1** and **2**, the lowest MIC values (4 and 1 µg/mL (16 and 5 µM), respectively) were found against the Gram-positive bacterium *Staphylococcus aureus* (Table 2). None of the compounds were found to be active against the two fungi tested in the concentration range investigated. Just as for the antibacterial properties of the compounds, compound **2** was the more potent compound in the cytotoxicity assay (IC_50_ for Huh7 cells: 112 µM and 11 µM for compounds **1** and **2**, respectively). The overall aim of the present study was to find new promising antibiotic compounds for future development into new antibiotic drugs. Compounds **1** and **2** have interesting spectra of antibacterial activity, and compound **1** has a IC_50_/MIC ratio indicating an acceptable therapeutic window. However, it is a grave problem that the more cytotoxic compound **2** is formed spontaneously from the less toxic **1**. This degradation may also affect the measured MIC and IC_50_ values for **1**. Additionally, compounds containing catechol moieties are known to be prone to oxidation, which indeed was found for compound **1** since it was spontaneously oxidized to compound **3** when left in acetone for a few days at room temperature. Thus, these compounds were considered unsuitable for further evaluation for development into future antibiotic drugs.

## 3. Materials and Methods

### 3.1. General Experimental Procedures

UV-spectra in MeOH were recorded on a Hitachi U-2001 spectrophotometer at room temperature. ^1^H and ^13^C NMR data were acquired at 303 K in acetone-*d*_6_ on a Bruker Avance III 600 MHz NMR spectrometer (Bruker Biospin GmBH, Rheinstetten, Germany) equipped with a 5-mm cryo-probe (^1^H, ^13^C, ^15^N, ^31^P). Standard pulse sequences supplied by Bruker were used for the determination of ^1^H and ^13^C chemical shifts and connectivities. For structure elucidation, 1D ^1^H, 1D ^13^C, COSY, DEPT-HSQC and HMBC were applied. Chemical shifts were determined relative to signals from residual acetone-*d*_5_ (δ_H_ 2.05) and acetone-*d*_6_ (δ_C_ 29.8). HPLC-MS was performed on an Agilent 1100 HPLC (Agilent, Palo Alto, CA, USA) connected to a maXis Impact ESI-QTOF MS (Bruker Daltonic GmbH., Bremen, Germany), and UHPLC-MS was done on an Agilent 1290 Infinity II connected to the same mass spectrometer. Preparative HPLC was performed on a Gilson 306/306 pump system (Gilson Inc., Middleton, WI, USA) with a Gilson 119 UV/VIS detector monitoring at 210 nm. Fractions were collected using a Gilson 204 fraction collector in 2.2-mL square well plates (Porvair Sciences Ltd., King’s Lynn, UK). MeCN of HPLC gradient grade (Sigma-Aldrich, St. Louis, MO, USA) and deionized filtered water (Millipore, Billerica, MA, USA) were used for mobile phases.

### 3.2. Strain Origin, Identity and Maintenance

The isolate of *P. spathulatum* strain Frisvad and Samson strain Em19 was isolated from a sample of Grottmans Bat Guano (Sneckenströms Ekohandel, Surte Sweden) purchased in Sweden but originating from the Philippines. The strain was isolated and purified on Malt Extract Agar [MEA, 15 g Malt Extract (BD Difco Ltd., Detroit, MI, USA), 15 g Bacto Agar (BD Difco Ltd.) in 1 L deionized water]. The strain Em19 was identified as *P. spatulathum* [15] by sequencing the internal transcribed spacer region (ITS1, 5.8S, ITS2; GenBank accession number MN759659) using the primers ITS1/ITS4 [30]. DNA was extracted and purified from fungal mycelium with the FastDNA spin kit for soil (MP Biomedicals/Fisher Scientific) according to the manufacturer’s instructions. Sequencing was done by Macrogen (EZ-seq, Macrogen Europe, Amsterdam, the Netherlands). The strain was initially maintained as an actively growing colony at MEA plate(s) in darkness at 20 °C and after first culturing as frozen/deep-frozen (−20 °C/−70 °C) stocks of ME grown mycelium/spores mixed with 20–25% glycerol. If needed, the strain was transferred to MEA plates for starting new cultures, otherwise new cultures were started directly from frozen stocks.

The strains of Escherichia coli LMG15862 (*E. coli*), Acinetobacter baumannii LMG1041T (*A. baumannii*), Enterobacter cloacae LMG2783T (*E. cloacae*), Klebsiella pneumoniae LMG20218 (*K. pneumoniae*), Pseudomonas aeruginosa LMG6395 (*P. aeruginosa*) and Staphylococcus aureus LMG15975 (*S. aureus*) were purchased from the Belgian Co-ordinated Collections of Micro-organisms, Gent, Belgium. The origin and cell/spore production of the strains of the yeast Candida albicans (*C.P. Robin*) Berkhout (*C. albicans*) strain H-29 and the fungus Aspergillus fumigatus Fres. strain J7 (A. fumigatus), was as previously described [31]. All strains were maintained as advised by the culture collections and cells/spores of all strains were stored at −70 °C.

### 3.3. Culture Conditions and Metabolite Sampling

During primary screening for antimicrobial activity, cultures of the strain Em19 (150 mL in 500 mL Erlenmeyer flasks) were grown in two liquid substrates: 1) Malt Extract [ME; 15 g Malt Extract (BD Difco Ltd.) in 1000 mL deionized H_2_O] and 2) in modified Mineral Medium pH 5.7 (MM-pH 5.7) for *Pseudomonas* [32,33] supplemented with 50 mL of Hagem Medium [34] [HM, 5 g ME (BD Difco Ltd.), 5 g glucose, 0.5 g KH_2_PO_4_, 0.5 g MgSO_4_ × 7 H_2_O, 0.5 g NH_4_Cl, 5 mL of Fe-EDTA stock (1.28 g FeSO_4_, 1.72 g EDTA, 1 L deionized water) in 1 L deionized water] and 250 mL Potato Dextrose Broth [PDB, 24 g Potato Dextrose Broth (BD Difco Ltd.)]. Potassium-sodium phosphate buffer (40 mL, 0.5 M, pH 5.7) was used to achieve the desired pH of the MM-pH 5.7 medium. The MM-pH 5.7 (700 mL), HM and PDB media were sterilized separately and prior to culturing, mixed to the MM-HMPDB-pH 5.7 medium composed of MM, HM, PDB and additionally supplemented with 2% glycerol. The 150 mL of ME cultures for prescreening were started by cutting around 20–25 small pieces from actively growing MEA Em19 culture and inoculating them into a ME containing Erlenmeyer flask. The MM-HMPDB-pH 5.7 cultures were started by transferring 10 mL of 72–96 h-old ME culture per 150 mL MM-HMPDB-pH 5.7.

For upscaling the production of selected active metabolites, the strain was cultured in MM-HMPDB-pH 5.7 (10 × 150 mL in 500 mL Erlenmeyer flasks) and to each culture, 10 mL of 72/96 h old ME start culture of Em19 was transferred for inoculation. Cultures were incubated on a rotary shaker (120/130 rpm) for 14–21 days at 15/20 °C in the dark.

To collect extracellular metabolites, one sterile nylon bag with the polymeric resin Amberlite XAD 16 (Sigma-Aldrich), approximately 8 g wet weight per bag, was added to each actively growing 150 mL culture, 72 to 96 h after inoculation.

For labeling with ^13^C labelled substrates, 18 mL MM-HMPDB-pH 5.7 in 125 mL Erlenmeyer flasks was inoculated with 2 mL of 72/96 h old ME start cultures of *P. spathulatum*. Prior to labelling, cultures were incubated on a rotary shaker (120 rpm) at 15 °C in the dark. After 96 h, 20 mg of 1-^13^C serine (CIL, Cambridge Isotope Laboratories Inc., Tewksbury, MA, USA), 1-^13^C cysteine (CIL), 1-^13^C tyrosine (CIL), ^13^CH_3_ methionine (CIL), sodium 1-^13^C pyruvate (Sigma-Aldrich, St. Louis, MO, USA), sodium 1-^13^C acetate (Sigma-Aldrich), or sodium 2-^13^C acetate (Sigma-Aldrich), was added to cultures together with one sterile nylon bag containing approximately 2 g wet weight Sepabeads^®^ SP850 (Mitsubishi Chemical Co., Tokyo, Japan). Em19 cultures were afterwards incubated on a rotary shaker (120 rpm) for 7–8 days at 15 °C in the dark, and then the adsorbent bags were washed with deionized H_2_O to remove fungal debris and extracted with 2 mL of MeOH prior to UHPLC-MS analysis (as below).

### 3.4. Isolation of Compounds **1** and **2**

The adsorbent bag was removed from a liquid culture of *P. spathulatum* (150 mL) after three weeks of cultivation. The bag was washed with deionized H_2_O, and then extracted with 2 × 10 mL of methanol and 2 × 10 mL MeCN. The extracts were pooled and dried under nitrogen and re-dissolved in 1.0 mL 50% MeCN, and fractionated by gradient reversed phase preparative HPLC (21.2 × 100 mm, 5 μm, Hypersil Gold, Thermo Scientific, Waltham, MA, USA) by applying a linear gradient of 10–95% MeCN in 10 min, followed by a 10 min hold at 95% aq. MeCN (flow 10 mL/min). Fractions were assayed for antibacterial activity and analysed by HPLC-MS as described below.

To obtain more of compounds **1** and **2**, ten liquid cultures of *P. spathulatum* (each 150 mL) were extracted using adsorbent bags (Amberlite XAD 16) as above. The combined and dried extracts were re-dissolved in 150 mL 20% MeCN and subsequently loaded onto 3 × 10 g C_18_ SPE columns. Each SPE column was washed with 50 mL 20% MeCN and then eluted with 50 mL 95% MeCN. The 95% MeCN extract was dried under reduced pressure, re-dissolved in 4 mL 50% MeCN and fractionated by gradient reversed phase preparative HPLC (as above). Fractions containing crude compound **1** and **2**, as indicated by HPLC-MS (as below), were individually pooled and dried under reduced pressure. The respective samples were dissolved in 1 mL eluent (as below) before further purification using reversed phase preparative HPLC (column and flow as above) at isocratic conditions with aqueous 30% and 40% MeCN, respectively. Following an analysis by HPLC-MS (as below), fractions containing compounds **1** and **2** were pooled and dried to yield 6.3 mg of **1** and 3.5 mg of **2**.

*Spathullin A (**1**, 6,7-dihydroxy-5,10-dihydropyrrolo[1,2-b]isoquinoline-3-carboxylic acid):* Spathullin A was obtained as white amorphous solid. UV λ_max_ (MeOH) nm (log ε): 206, 274 (4.45, 4.11); NMR-data, see Table 1; HRMS: *m*/*z* 246.0762 [M + H]^+^ (calcd. for C_13_H_12_NO_4_, 246.0761).

*Spathullin B (**2**, 5,10-dihydropyrrolo[1,2-b]isoquinoline-6,7-diol):* Spathullin B was obtained as colorless oil. UV λ_max_ (MeOH) nm (log ε) 206, 282 (4.48, 3.49); NMR-data, see Table 1; HRMS: *m*/*z* 202.0861 [M + H]^+^ (calcd. for C_12_H_12_NO_2_, 202.0863).

*Spathullin C (**3**, 6,7-dioxo-6,7-dihydropyrrolo[1,2-b]isoquinoline-3-carboxylic acid):* Spathullin C was obtained as purple amorphous solid. NMR-data, see Table 1; HRMS: *m*/*z* 242.0451 [M + H]^+^ (calcd. for C_13_H_8_NO_4_, 242.0448).

### 3.5. In Vitro Bioassay

Chromatographic fractions during primary screening were assayed for antimicrobial activity using an earlier developed protocol (“Microtiter plate assay 2”) [35], which is based on the inhibition of cell growth or spore germination in microtiter plates. The following organisms were used for the bioassays: *E. coli*, *A. baumannii*, *E. cloacae*, *K. pneumoniae*, *P. aeruginosa*, *S. aureus*, *C. albicans* and *A. fumigatus*. Aliquots of HPLC fractions were transferred to 96-well microtiter plates and the solvent was evaporated in a fume-hood overnight. Cell suspensions (all bacterial strains and *C. albicans*) or spore suspensions (*A. fumigatus*), 100 μL at a concentration of 10^4^ cells/spores per mL, in VBP for bacteria, ME for *A. fumigatus*, and Yeast Extract—Malt Extract Medium, ISP2 [36], (4 g Yeast Extract (BD Difco Ltd.), 10 g Malt Extract (BD Difco Ltd.), 4 g dextrose in 1 L deionized water) for *C. albicans*, were added to the wells and incubated at 37 °C in the dark for 16 to 24 h. The effect on the bacterial/fungal growth was estimated visually according to the following scale: 3—full growth inhibition, 2—intermediate growth inhibition, 1—weak growth inhibition, 0—no growth inhibition. Positive controls were cell/spore suspensions only, and sterile medium was used as negative control. Further steps of purification of compounds **1** and **2** were done with *S. aureus* as the activity indicator.

### 3.6. Analysis by HPLC-MS and UHPLC-MS

Culture extracts and HPLC fractions were analyzed by HPLC-MS on a reversed phase HPLC column (3.0 × 50 mm, 2.6 μm, Accucore RP-MS, Thermo Scientific, Waltham, MA, USA), using a gradient of MeCN in water (10–95% MeCN in 3 min, 95% MeCN for 4 min, at 0.8 mL/min, with 0.2% formic acid). ^13^C-labelling experiments were analyzed by UHPLC-MS on a reversed phase UHPLC column (2.1 × 50 mm, 1.5 μm, Thermo Accucore Vanquish RP-MS, Thermo Scientific, Waltham, MA, USA), using a gradient of MeCN in water (10–95% MeCN in 3 min, 95% MeCN for 1.2 min, at 0.9 mL/min, with 0.2% formic acid). For both HPLC-MS and UHPLC-MS, the mass spectrometer was operated in positive mode with scanning of *m*/*z* 50–1500, and calibration of the mass spectra was obtained by using sodium formate clusters.

### 3.7. MIC Determination

The MIC of all compounds was measured by means of a broth micro-dilution method in 96-well microtiter plates using all organisms used in the in vitro bioassay. The test media were AM3 Broth (BD Difco Ltd.) mixed with phosphate buffered saline (PBS, Amresco LLC, Solon, OH, USA) in 1:4 ratio (*E. coli*, *A. baumannii*, *E. cloacae*, *K. pneumoniae* and *P. aeruginosa*), AM3 Broth mixed with PBS in 1:1 ratio (*S. aureus*), ME (*A. fumigatus*) and for *C. albicans* ISP2. Prior to MIC-tests, the cell concentration of tested bacterial/fungal pathogens was adjusted to between 1 and 5 × 10^5^ cells/spores per mL. Cell concentration was confirmed by colony counting after diluting and plating the respective bacterial pathogen on Vegetable Peptone Broth agar plates (VPA, 10 g Vegetable Peptone Broth (Oxoid Ltd., Basingstoke, UK), 15 g Bacto Agar (Difco Ltd., Tyrone) in 1 L deionised water) directly after each of MIC-tests. The cells of *C. albicans* were plated on ISP2 agar plates (ISP2, 15 g Bacto Agar (Difco Ltd.) in 1 L deionised water) and respectively spores of *A. fumigatus* were plated on MEA plates. Plates were subsequently incubated at 37 °C in the dark, and viable colonies counted after 16 to 24 h. To estimate MIC values (concentration range between 0.05 and 50 µg/mL), solutions of the tested compounds (100, 10 and 1 and if needed 0.1 μg/mL in MeOH) were dispensed into wells of microtiter plate(s) and the solvent was evaporated in a fume-hood. Subsequently, suspensions of pathogen cells in the appropriate medium were dispensed to the wells of microtiter plates and the growth of pathogens was observed after 16 to 20 h of incubation at 37 °C in the dark. Positive controls were the respective pathogen cells suspended in untreated sterile media without addition of compounds(s) and negative controls were the media only. MIC values were defined as the lowest concentration of each compound with no visible growth of pathogen. All MIC tests were performed in triplicate and repeated at least twice.

### 3.8. Determination of Toxicity

Huh-7 cells (hepatocellular carcinoma cells, ReBLikon GmbH, Schriesheim, Germany) were passaged into 96-well microplates (2 × 10^4^ cells/well) followed by the addition of the test substances the next day, in duplicate samples. The number of viable cells was determined after 48 h by using a soluble formazan (XTT) assay [37] and the compound troxacitabine was used as positive control.

## Figures and Tables

**Table 1:** NMR data for compounds **1**–**3** (acetone-*d*_6_, 600 and 150 MHz, 303 K).

	1		2		3	
Pos.	δ_C_	δ_H_, mult. (J in Hz)	δ_C_	δ_H_, mult. (J in Hz)	δ_C_	δ_H_, mult. (J in Hz)
1	105.5	6.02, d (4.0)	103.7	5.83, m	108.8	6.92, d (4.3)
2	119.0	6.97, d (4.0)	108.6	6.04, dd (3.4, 2.7)	125.8	7.64, d (4.3)
3	121.6	-	118.9	6.76, dd (2.7, 1.7)	120.8	-
5	43.4	5.54, s	43.4	5.05, br s	134.2	10.16, s
5a	120.6	-	121.0	-	119.6	-
6	142.7	-	142.6	-	179.1	-
7	143.5	-	143.3	-	181.4	-
8	115.0	6.81, d (8.1)	114.9	6.77, d (8.0)	129.3	6.48, d (10.0)
9	119.2	6.67, d (8.1)	119.4	6.65, d (8.0)	144.5	7.71, d (10.0)
9a	124.7	-	126.3	-	126.7	-
10	28.4	3.97, s	28.1	3.87, br s	121.4	7.85, s
10a	136.9	-	128.2	-	137.8	-
11	162.4	-	-	-	n.d.^a^	-

^a^ n.d.: not detected.

**Table 2:** Minimal inhibitory concentrations (MIC) against bacteria and fungi for compounds **1** and **2**, compared to MIC for Meropenem (MEM) and Ciprofloxacin (CIP).

	MIC (μg/mL)			
Test Organisms	1	2	MEM	CIP
*Escherichia coli* LMG15862	15	5	0.5	0.06
*Acinetobacter baumannii* LMG1041T	15	5	2	1
*Enterobacter cloacae* LMG2783T	15	5	1	0.03
*Klebsiella pneumonia* LMG20218	64	32	1	0.5
*Pseudomonas aeruginosa* LMG6395	>64	64	1	0.25
*Staphylococcus aureus* LMG15975	4	1	0.5	0.25
*Candida albicans* H-29	>64	>64	n.t.^a^	n.t.
*Aspergillus fumigatus* J7	>64	>64	n.t.	n.t.

^a^ not tested.

## Supplementary Materials

The following are available online, Figure S1: ^1^H NMR (acetone-*d*_6_, 600 MHz) spectrum of **1**. Figure S2: ^13^C NMR (acetone-*d*_6_, 150 MHz) spectrum of **1**. Figure S3: COSY NMR (acetone-*d*_6_) spectrum of **1**. Figure S4: HSQC NMR (acetone-*d*_6_) spectrum of **1**. Figure S5: HMBC NMR (acetone-*d*_6_) spectrum of **1**. Figure S6: ^1^H NMR (acetone-*d*_6_, 600 MHz) spectrum of **2**. Figure S7: ^13^C NMR (acetone-*d*_6_, 150 MHz) spectrum of **2**. Figure S8: COSY NMR (acetone-*d*_6_) spectrum of **2**. Figure S9: HSQC NMR (acetone-*d*_6_) spectrum of **2**. Figure S10: HMBC NMR (acetone-*d*_6_) spectrum of **2**. Figure S11: ^1^H NMR (acetone-*d*_6_, 600 MHz) spectrum of **3**. Integral values shown for signals from compound **3**, the other signals belong to compound **1**, the solvent or residual amounts of methanol. Figure S12: ^13^C NMR (acetone-*d*_6_, 150 MHz) spectrum of **3**. Shift values shown for carbons from compound **3**, remaining signals belong to compound **1**. Figure S13: COSY NMR (acetone-*d*_6_) spectrum of **3**. Figure S14: HSQC NMR (acetone-*d*_6_) spectrum of **3**. Figure S15: HMBC NMR (acetone-*d*_6_) spectrum of **3**. Figure S16: HRMS base peak chromatogram of the mixture of compound **3** (2.6 min—*m*/*z* 242.0451) and compound **1** (3.0 min—*m*/*z* 246.0762). Figure S17: HR mass spectrum of compound **1**, *m*/*z* 246.0762 [M + H]^+^ (calcd. for C_13_H_12_NO_4_, 246.0761). Figure S18: HR mass spectrum of compound **2**, *m*/*z* 202.0861 [M + H]^+^ (calcd. for C_12_H_12_NO_2_, 202.0863). Figure S17: HR mass spectrum of compound **3**, *m*/*z* 242.0451 [M + H]^+^ (calcd. for C_13_H_8_NO_4_, 242.0448).
