# Supplementary material for: Antibacterial Isoquinoline Alkaloids from the Fungus Penicillium Spathulatum Em19
Source: Molecules. 2019 Dec 17;24(24):4616. doi: 10.3390/molecules24244616 (PMC6943532; doi:10.3390/molecules24244616)
Supplement: Supplementary file 1 [file molecules-24-04616-s001.pdf]

Supplementary Materials

Antibacterial isoquinoline alkaloids from the fungus *Penicillium spathulatum* Em19

Christina Nord [1], Jolanta J. Levenfors [1,2], Joakim Bjerketorp [1,2], Christer Sahlberg [3], Bengt Guss [4], Bo Öberg [2,5], and Anders Broberg [1,*]

[1] Department of Molecular Sciences, Uppsala BioCentrum, Swedish University of Agricultural Sciences, P.O. Box 7015, SE-750 07 Uppsala, Sweden; Christina.Nord@slu.se (C.N.), Jolanta.Levenfors@slu.se (J.J.L.), Joakim.Bjerketorp@slu.se (J.B.), Anders.Broberg@slu.se (A.B.)
[2] Ultupharma AB, Södra Rudbecksgatan 13, SE-752 36 Uppsala, Sweden. bo.oberg1@gmail.com
[3] Medivir AB, P.O. Box 1086, SE-141-22 Huddinge, Sweden; christer.sahlberg@gmail.com
[4] Department of Biomedical Sciences and Veterinary Public Health, Swedish University of Agricultural Sciences, P.O. Box 7036, SE-750 07 Uppsala, Sweden; Bengt.Guss@slu.se
[5] Department of Medicinal Chemistry, Uppsala University, P.O. Box 574, SE-751 23 Uppsala, Sweden
[*] Correspondence: Anders.Broberg@slu.se; Tel.: +46 18 672217.

Contents

Figure S1: ^{1}H NMR (acetone-d_6, 600 MHz) spectrum of **1**. 3
Figure S2: ^{13}C NMR (acetone-d_6, 150 MHz) spectrum of **1**. 4
Figure S3: COSY NMR (acetone-d_6) spectrum of **1**. 5
Figure S4: HSQC NMR (acetone-d_6) spectrum of **1**. 6
Figure S5: HMBC NMR (acetone-d_6) spectrum of **1**. 7
Figure S6: ^{1}H NMR (acetone-d_6, 600 MHz) spectrum of **2**. 8
Figure S7: ^{13}C NMR (acetone-d_6, 150 MHz) spectrum of **2**. 9
Figure S8: COSY NMR (acetone-d_6) spectrum of **2**. 10
Figure S9: HSQC NMR (acetone-d_6) spectrum of **2**. 11
Figure S10: HMBC NMR (acetone-d_6) spectrum of **2**. 12
Figure S11: ^{1}H NMR (acetone-d_6, 600 MHz) spectrum of **3**. Integral values shown for signals from compound 3, the other signals belong to compound **1**, the solvent or residual amounts of methanol. 13
Figure S12: ^{13}C NMR (acetone-d_6, 150 MHz) spectrum of **3**. Shift values shown for carbons from compound 3, remaining signals belong to compound **1**. 14
Figure S13: COSY NMR (acetone-d_6) spectrum of **3**. 15
Figure S14: HSQC NMR (acetone-d_6) spectrum of **3**. 16

Figure S15: HMBC NMR (acetone-d_6) spectrum of **3**....17

Figure S16: HRMS base peak chromatogram of the mixture of compound **3** (2.6 min – m/z 242.0451) and compound **1** (3.0 min – m/z 246.0762). 18

Figure S17: HR mass spectrum of compound **1**, m/z 246.0762 $[M+H]^+$ (calcd. for $C_{13}H_{12}NO_4$, 246.0761). 18

Figure S18: HR mass spectrum of compound **2**, m/z 202.0861 $[M+H]^+$ (calcd. for $C_{12}H_{12}NO_2$, 202.0863). 18

Figure S17: HR mass spectrum of compound **3**, m/z 242.0451 $[M+H]^+$ (calcd. for $C_{13}H_8NO_4$, 242.0448). 18

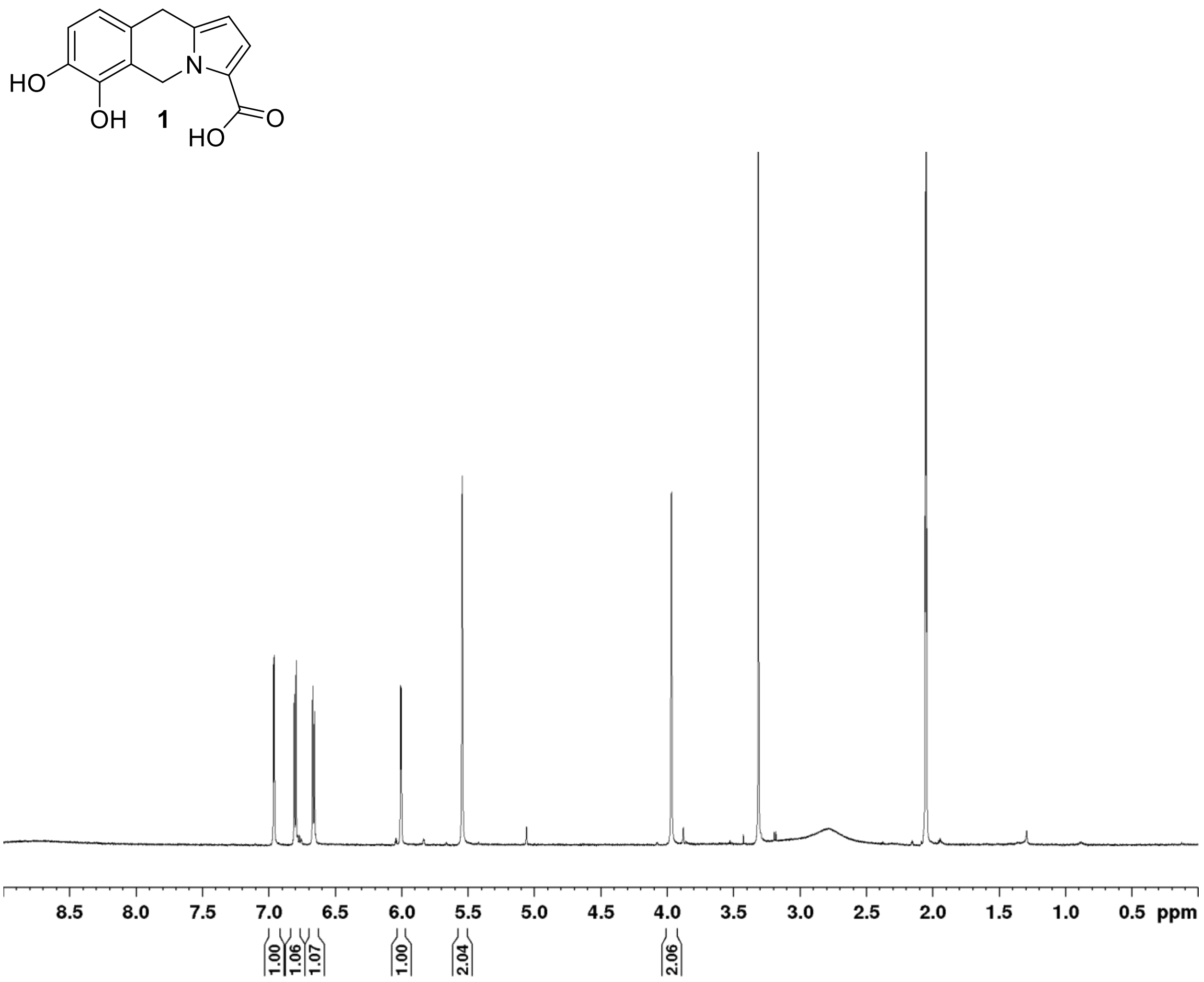

Figure S1: ^{1}H NMR (acetone-d_6, 600 MHz) spectrum of **1**. Signal at δ_H 2.05 is acetone-d_5 and the signal at δ_H 3.31 is methanol.

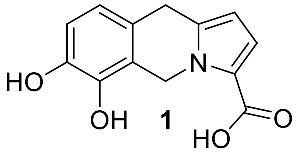

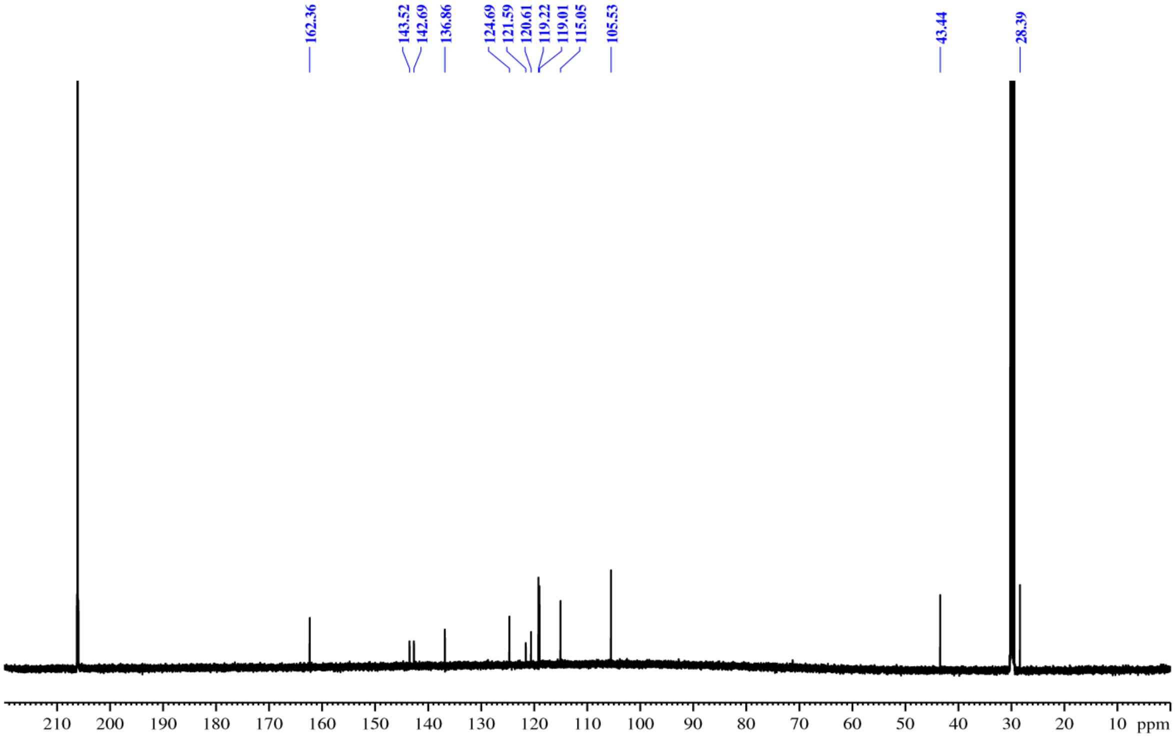

Figure S2: ^{13}C NMR (acetone-d_6, 150 MHz) spectrum of **1**.

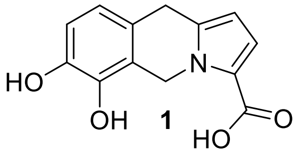

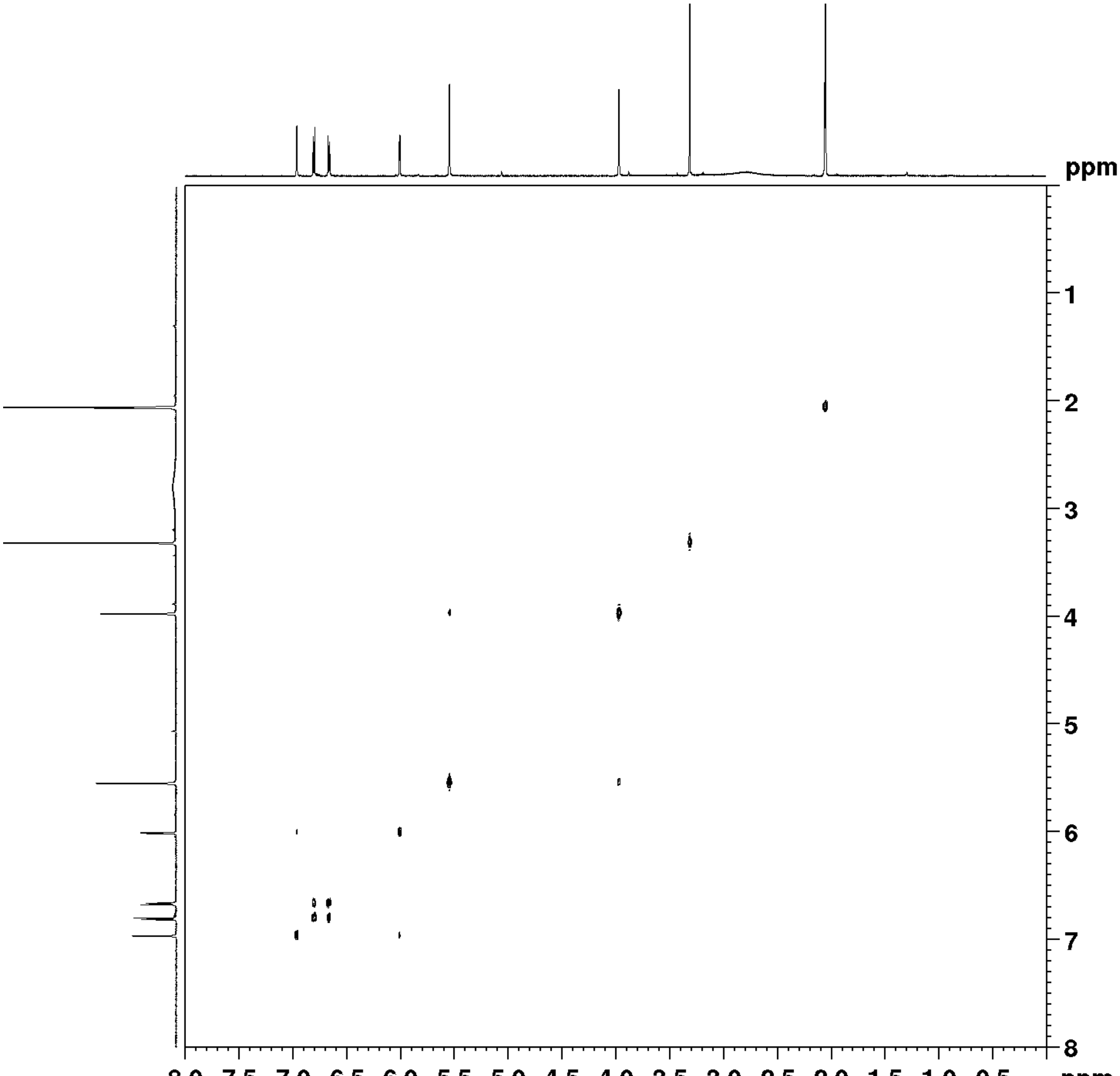

Figure S3: COSY NMR (acetone-d_6) spectrum of **1**. Signal at δ_H 2.05 is acetone-d_5 and the signal at δ_H 3.31 is methanol.

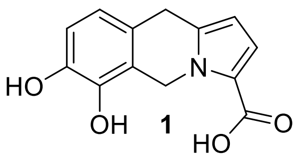

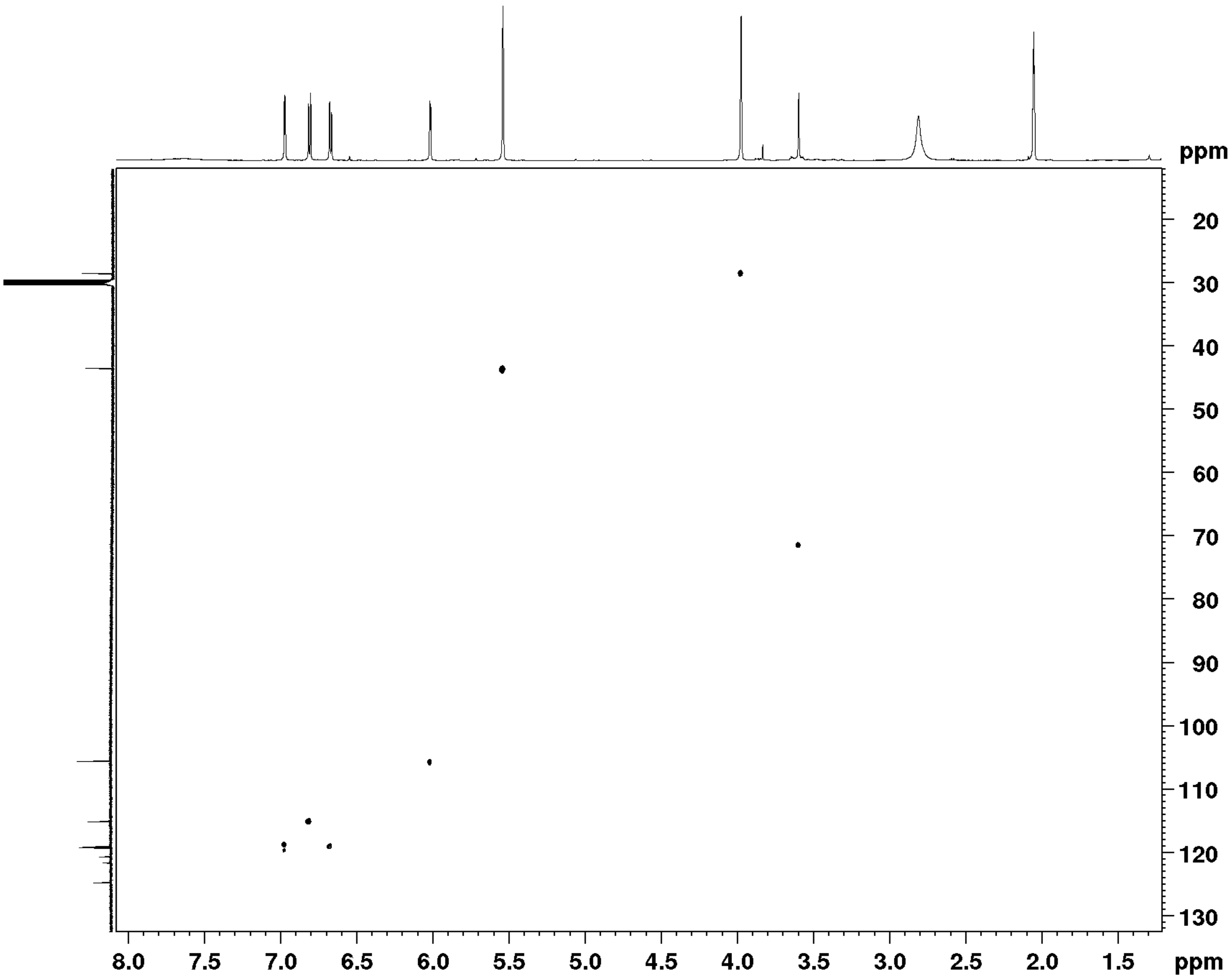

Figure S4: HSQC NMR (acetone-d_6) spectrum of **1**. Signal at δ_H 3.59/δ_C 71.4 is from a polyethylene glycol type contaminant.

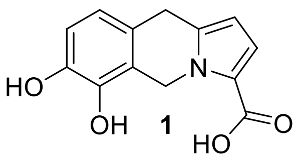

1

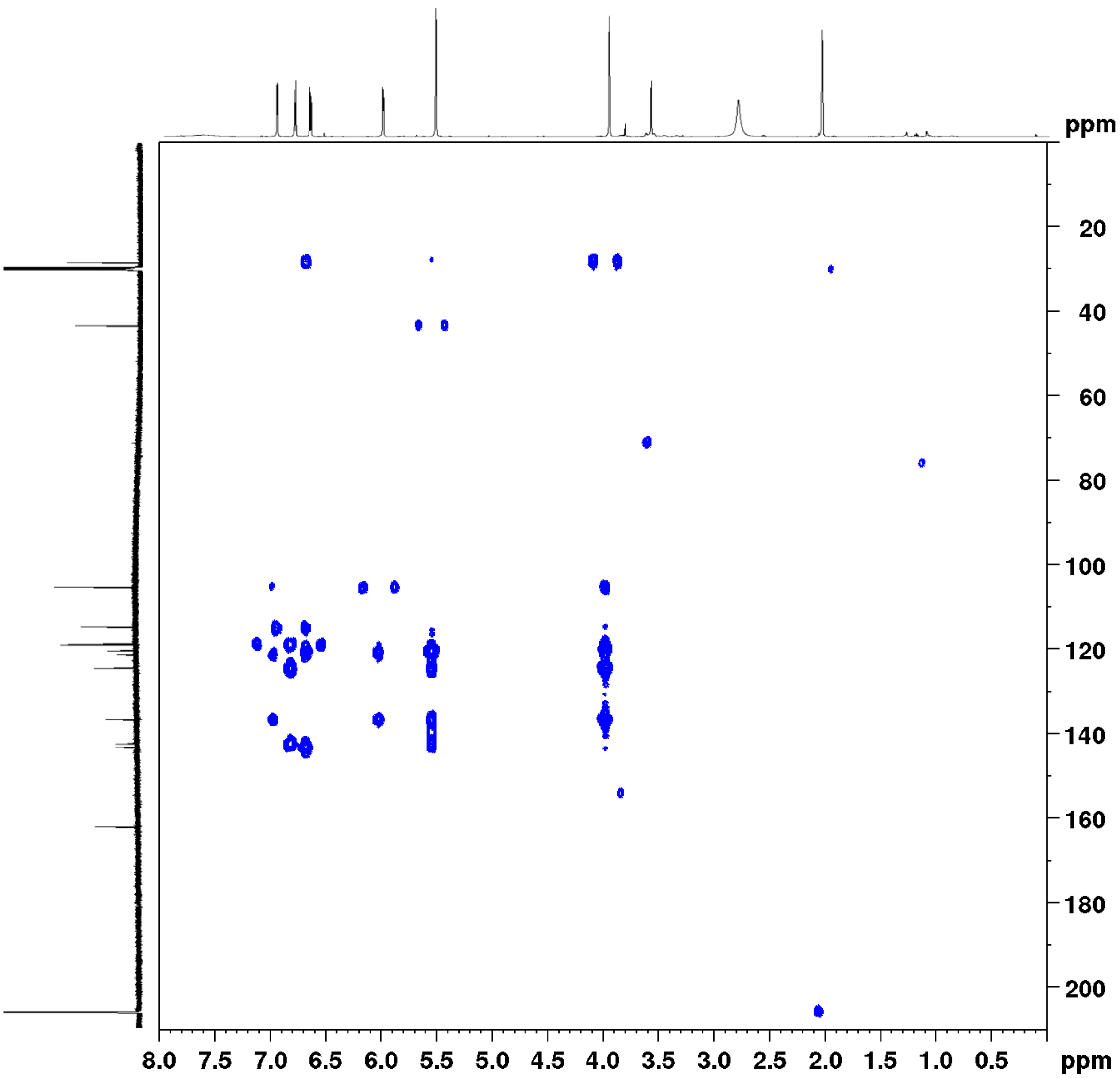

Figure S5: HMBC NMR (acetone-d_6) spectrum of **1**. Signal at δ_H 3.59/δ_C 71.4 is from a polyethylene glycol type contaminant.

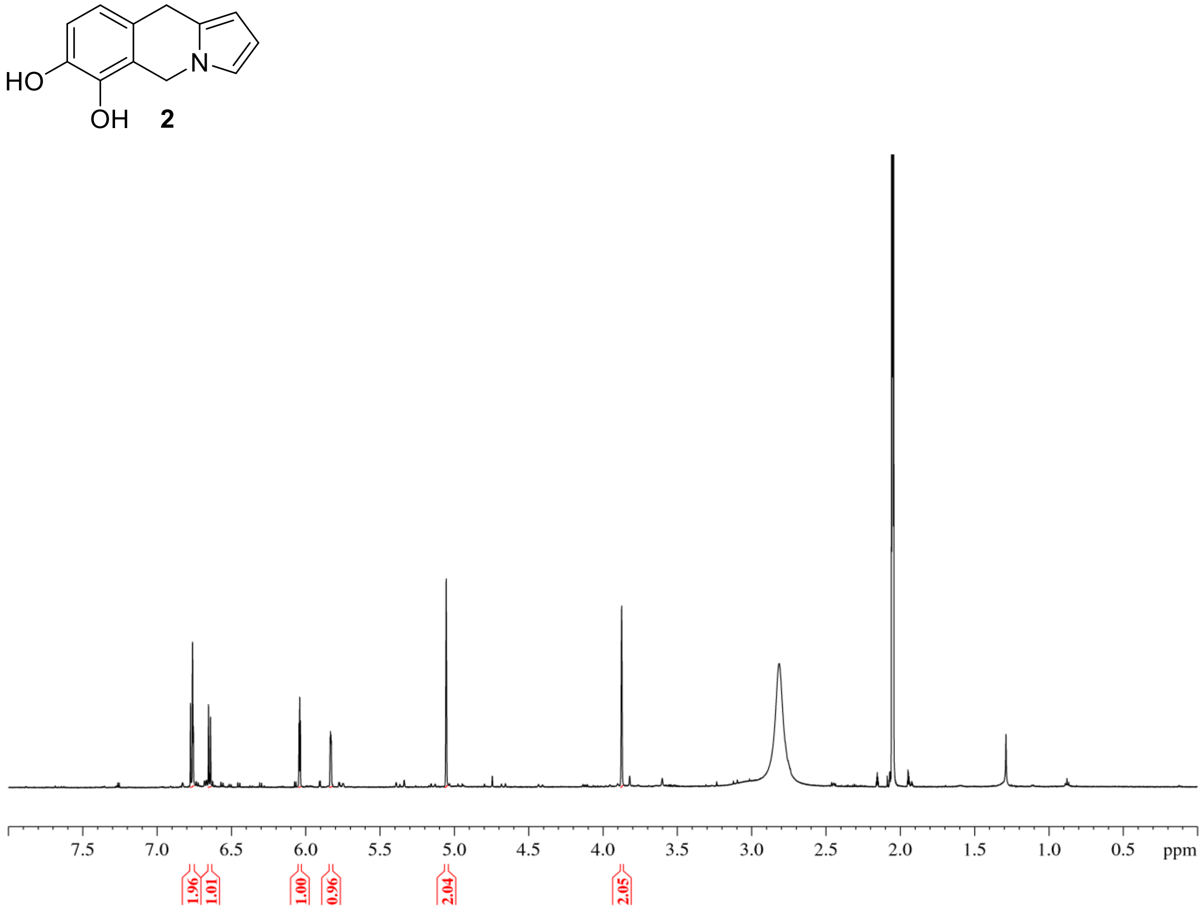

Figure S6: ^{1}H NMR (acetone-d_6, 600 MHz) spectrum of **2**. Signal at δ_H 2.05 from acetone-d_5.

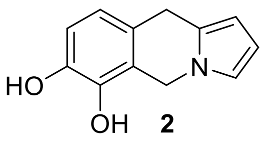

2

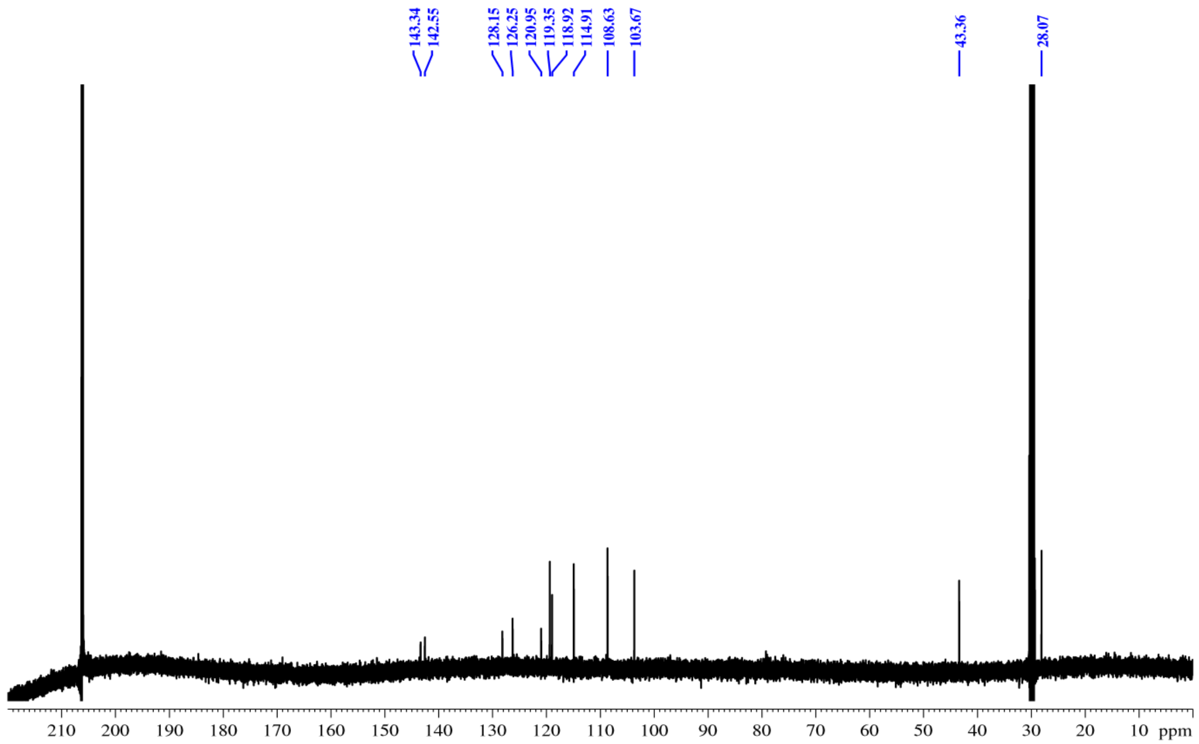

Figure S7: ^{13}C NMR (acetone-d_6, 150 MHz) spectrum of **2**.

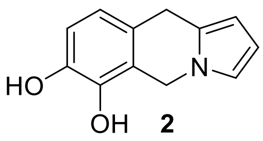

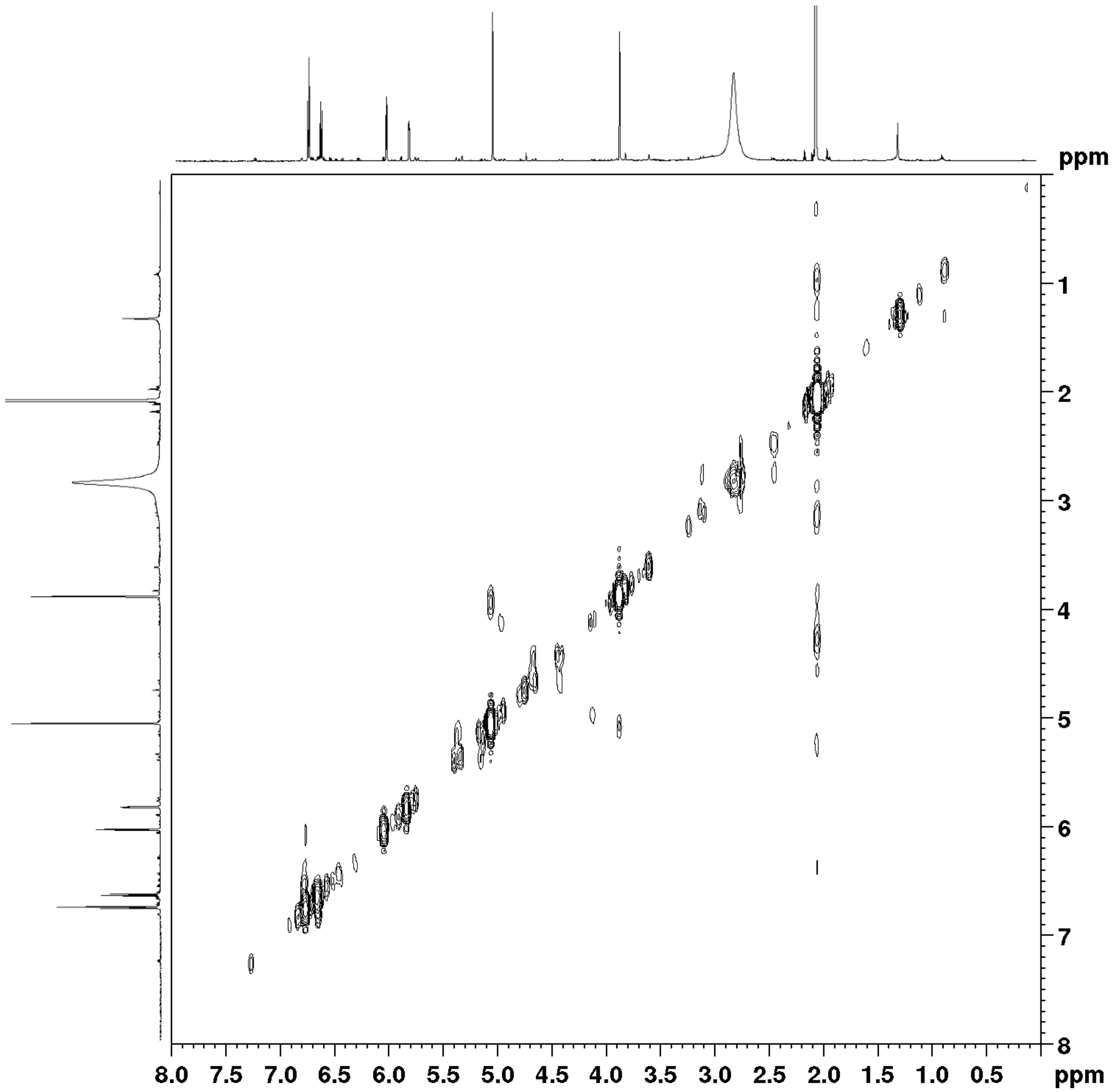

Figure S8: COSY NMR (acetone-d_6) spectrum of **2**.

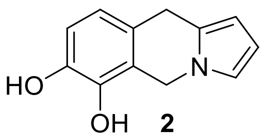

2

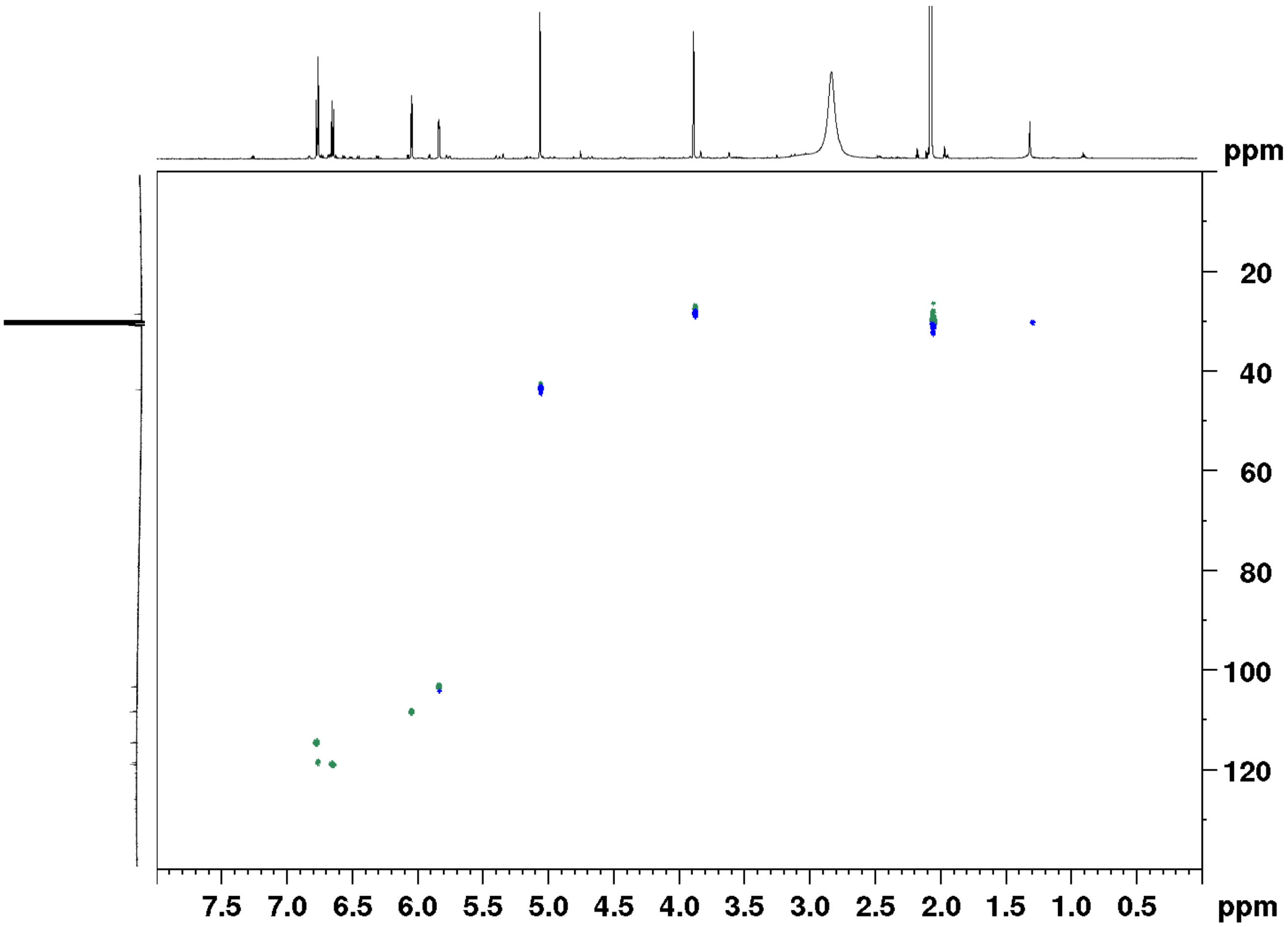

Figure S9: HSQC NMR (acetone-d_6) spectrum of **2**.

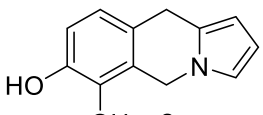

2

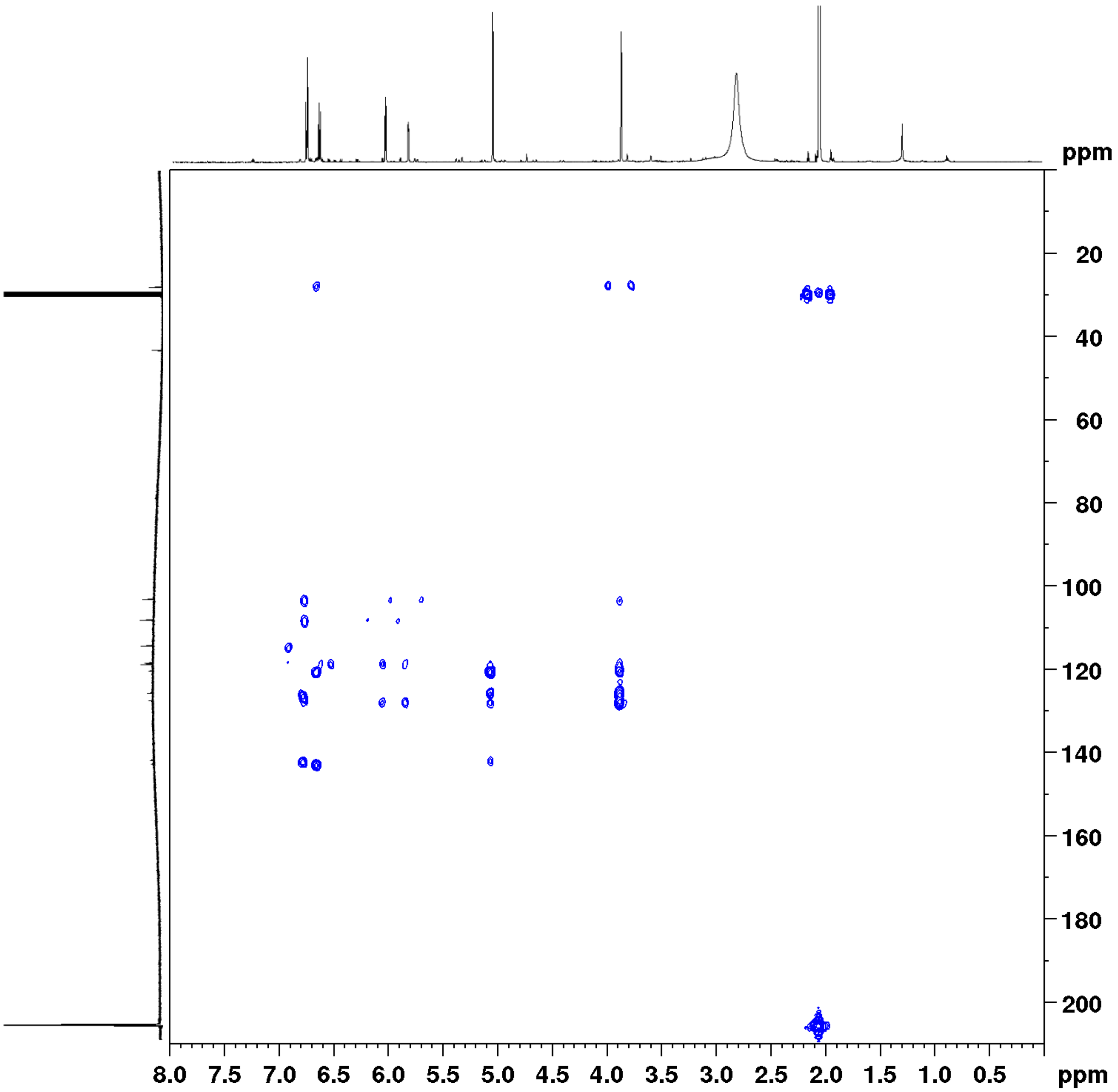

Figure S10: HMBC NMR (acetone-d_6) spectrum of **2**.

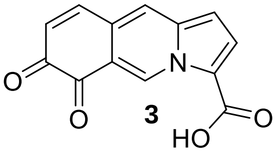

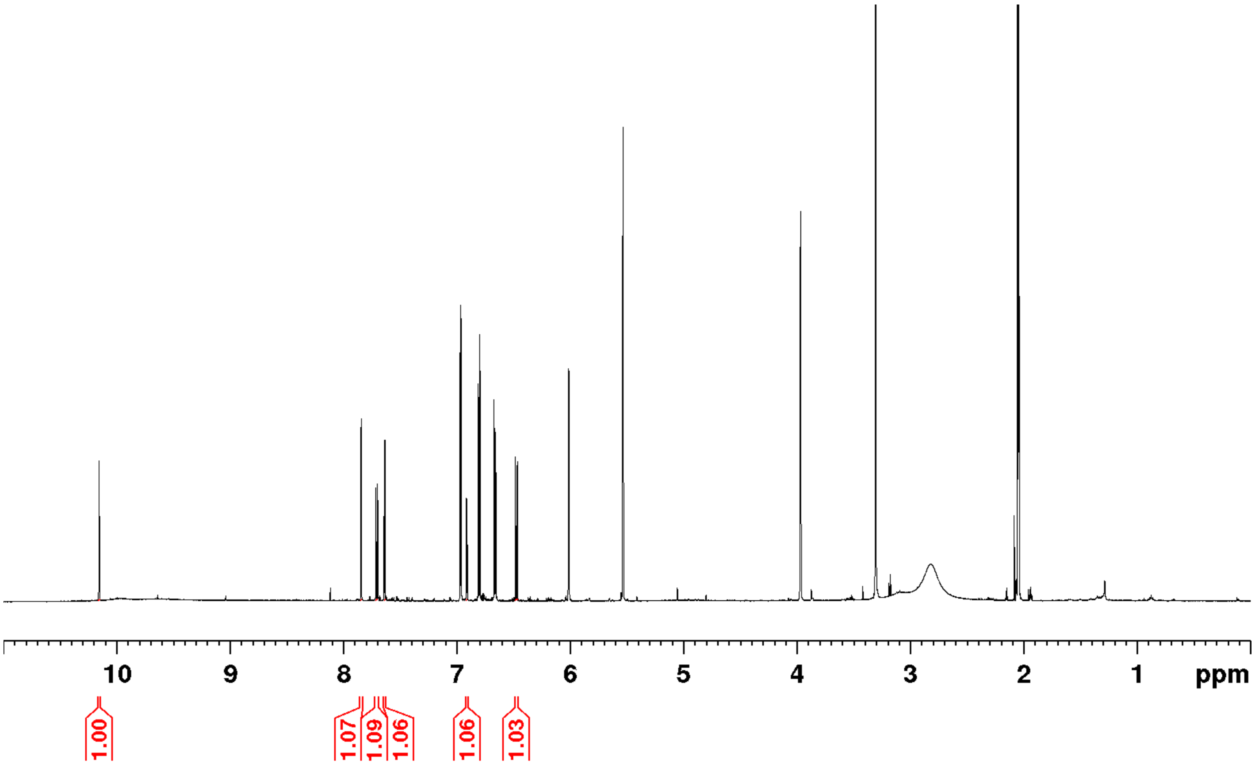

Figure S11: ^{1}H NMR (acetone-d_6, 600 MHz) spectrum of **3**. Integrals are shown for signals from compound **3**, the other signals belong to compound **1**, acetone-d_5 (δ_H 2.05) or residual methanol (δ_H 3.31).

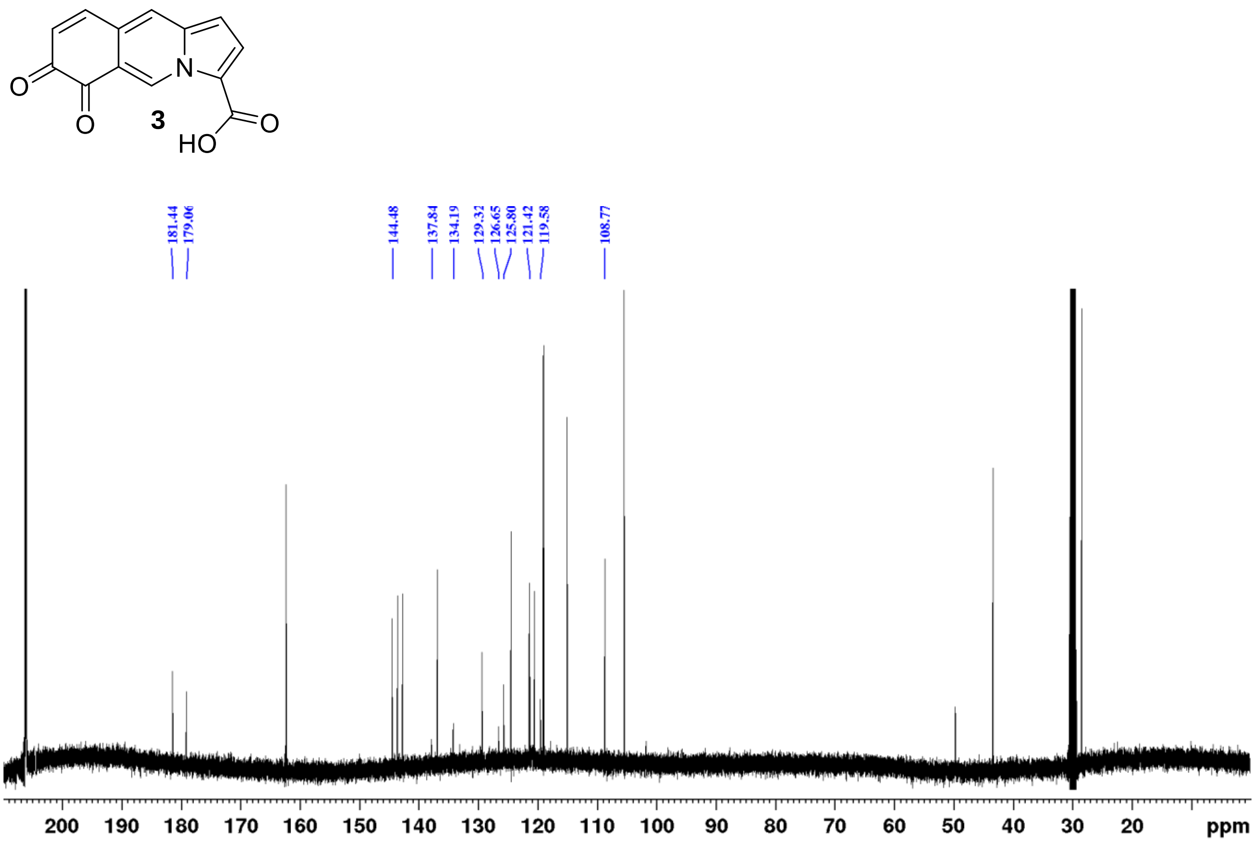

Figure S12: ^{13}C NMR (acetone-d_6, 150 MHz) spectrum of **3**. Chemical shifts are shown for carbons from compound **3**, and remaining signals belong to compound **1**.

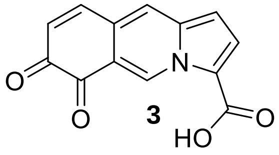

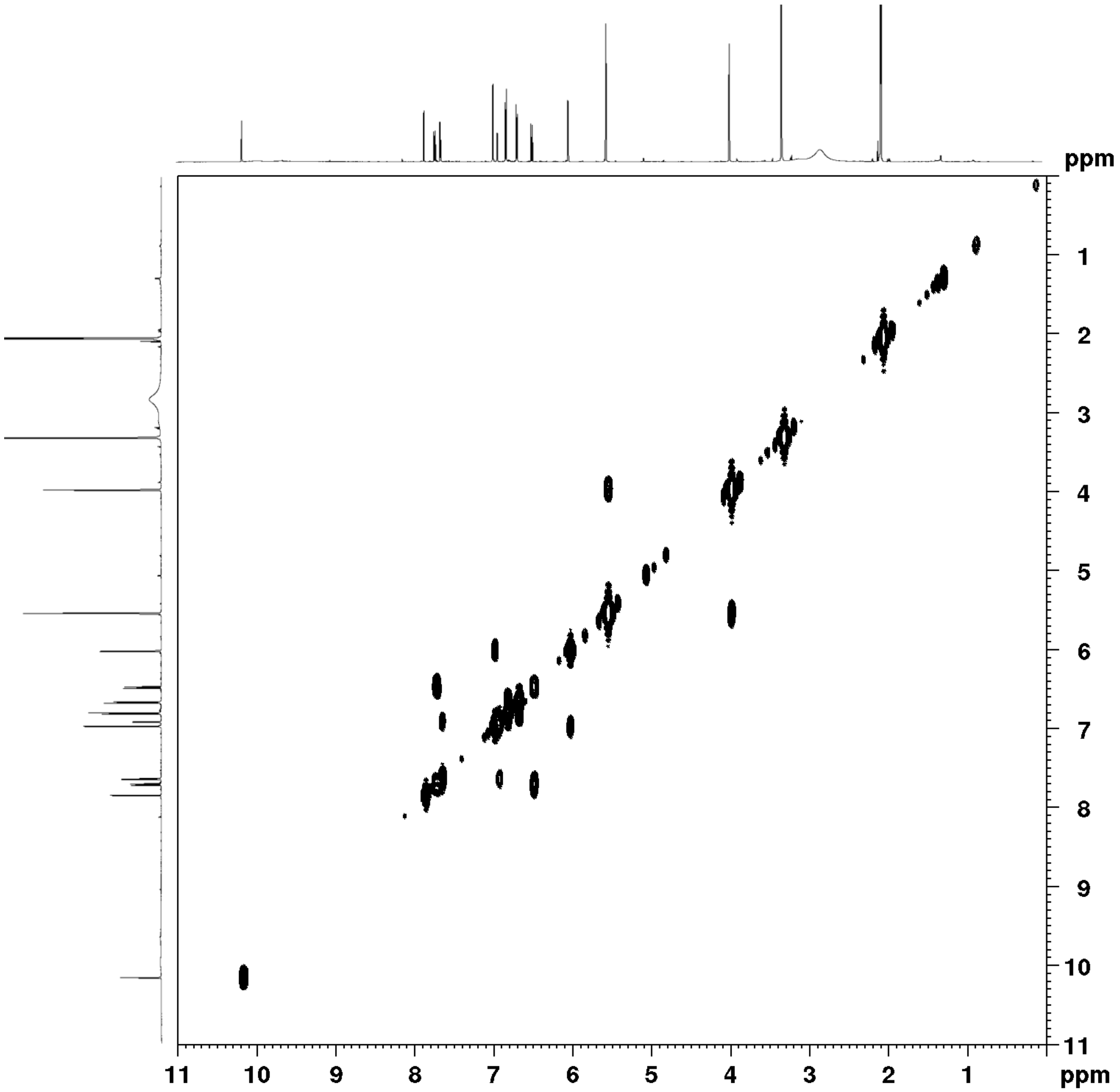

Figure S13: COSY NMR (acetone-d_6) spectrum of **3**.

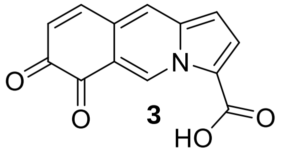

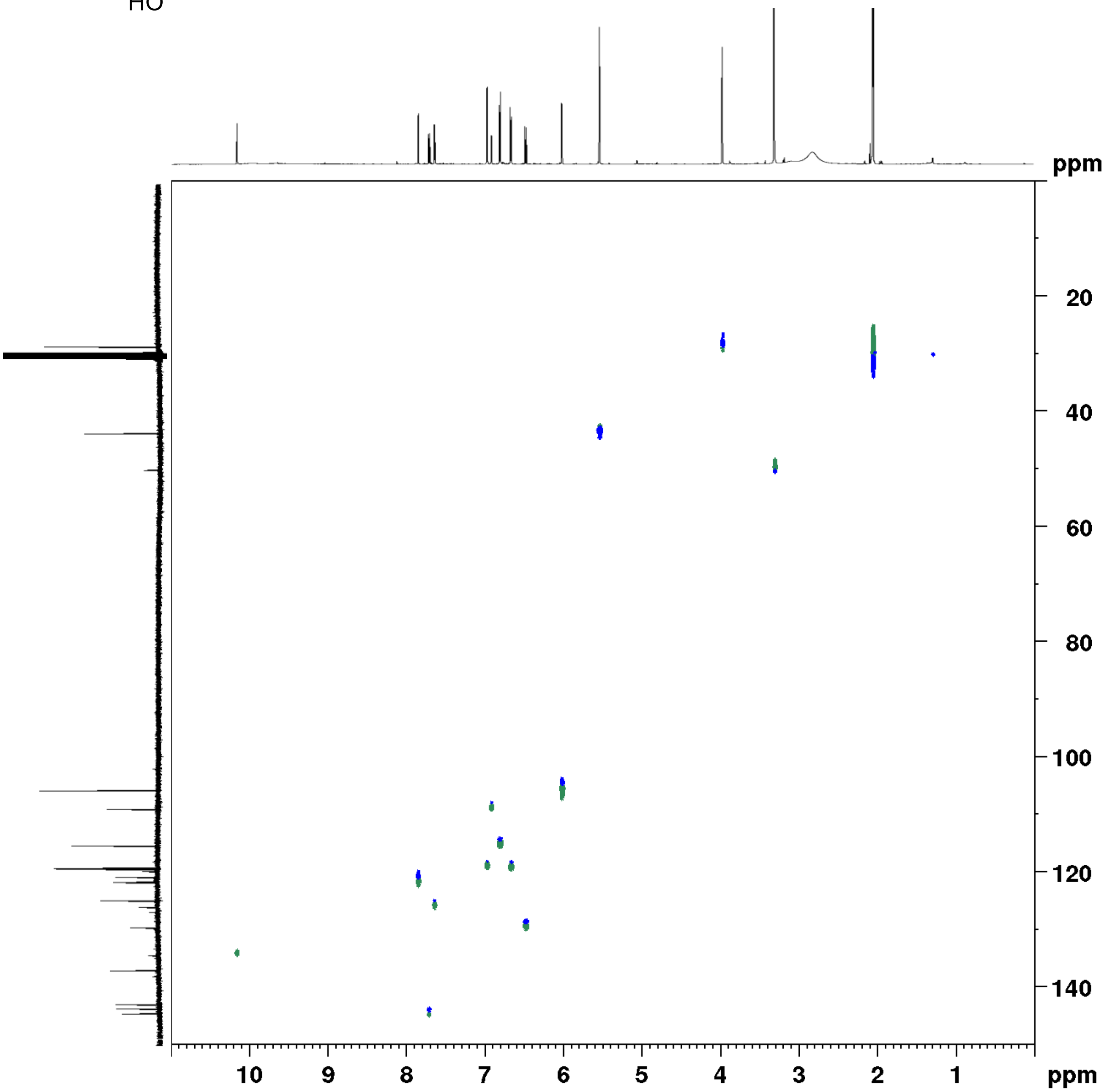

Figure S14: HSQC NMR (acetone-d_6) spectrum of **3**.

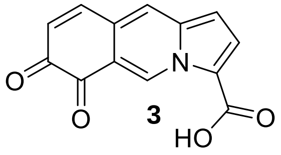

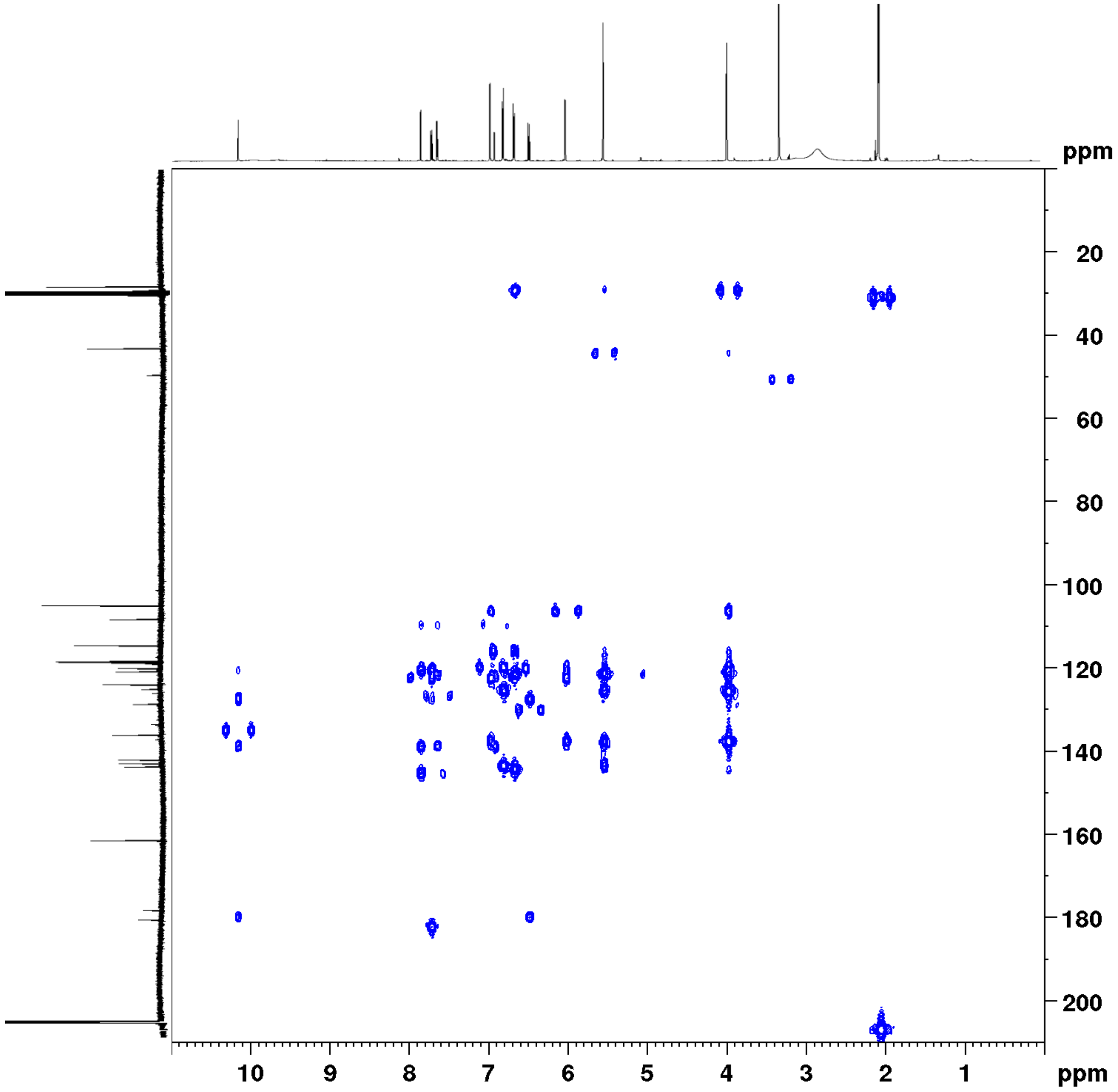

Figure S15: HMBC NMR (acetone-d_6) spectrum of **3**.

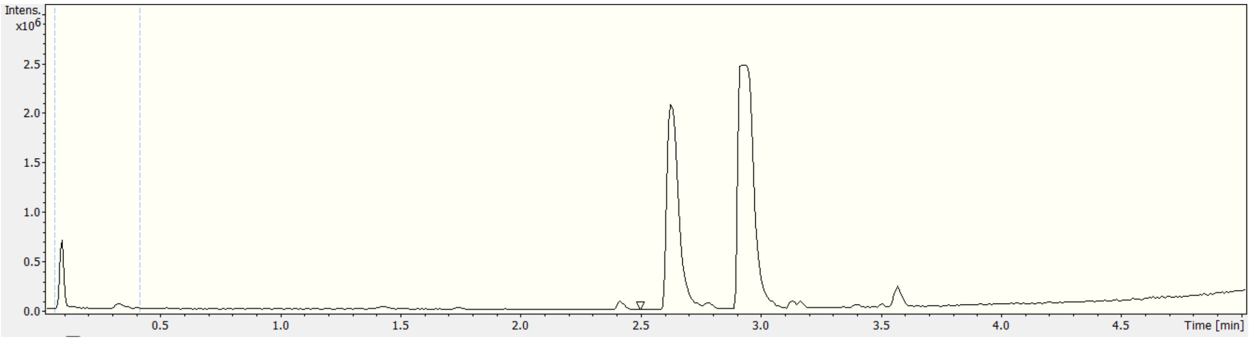

Figure S16: HRMS base peak chromatogram of the mixture of compound **3** (2.6 min – *m/z* 242.0451) and compound **1** (3.0 min – *m/z* 246.0762).

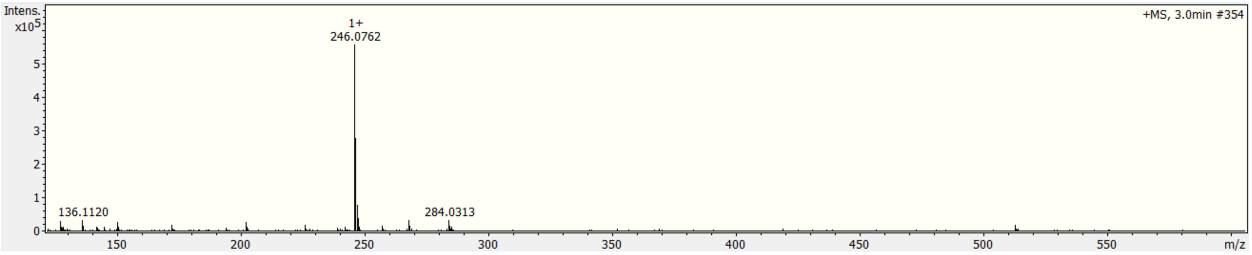

Figure S17: HR mass spectrum of compound **1**, *m/z* 246.0762 $[M+H]^+$ (calcd. for $C_{13}H_{12}NO_4$, 246.0761).

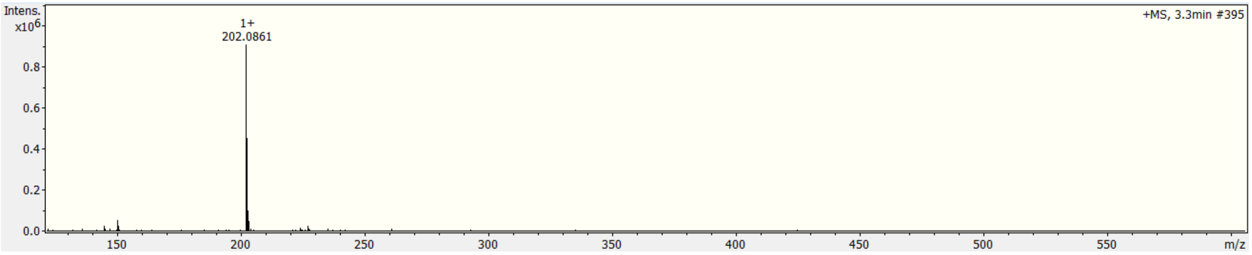

Figure S18: HR mass spectrum of compound **2**, *m/z* 202.0861 $[M+H]^+$ (calcd. for $C_{12}H_{12}NO_2$, 202.0863).

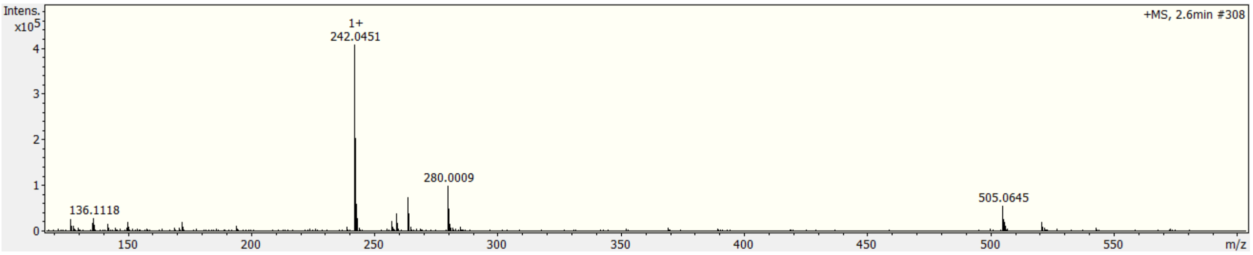

Figure S19: HRMS spectrum of compound **3**, *m/z* 242.0451 $[M+H]^+$ (calcd. for $C_{13}H_8NO_4$, 242.0448).
